# Evaluation of Behavioral and Pharmacological Effects of Hydroalcoholic Extract of *Valeriana prionophylla* Standl. from Guatemala

**DOI:** 10.1155/2011/312320

**Published:** 2011-06-30

**Authors:** Iandra Holzmann, Valdir Cechinel Filho, Ticiana C. Mora, Armando Cáceres, Jose Vicente Martínez, Sully M. Cruz, Márcia Maria de Souza

**Affiliations:** ^1^Programa de Mestrado em Ciências Farmacêuticas and Núcleo de Investigações Químico-Farmacêuticas (NIQFAR)/CCS, Universidade do Vale do Itajaí (UNIVALI), 88302202 Itajaí, SC, Brazil; ^2^Facultad de Ciencias Químicas y Farmacia, Universidad de San Carlos de Guatemala (USAC), 01012, Guatemala; ^3^Facultad de Agronomía, Universidad de San Carlos de Guatemala (USAC), 01012, Guatemala

## Abstract

There are few studies on the pharmacological properties of *Valeriana prionophylla* Standl. (VP), known as “Valeriana del monte”, and used in Mesoamerican folk medicine to treat sleep disorders. This study examines the pharmacological effects of the hydroalcoholic extract of the dry rhizome using the open field, rota rod, elevated plus-maze (EPM), forced swimming (FST), strychnine- and pentobarbital-induced sleeping time, PTZ-induced seizures, and the inhibitory avoidance tests. VP did not show any protective effect against PTZ-induced convulsions. In the EPM, exhibited an anxiolytic-like effect through the effective enhancement of the entries (38.5%) and time spent (44.7%) in the open arms, when compared with control group. Time spent and the numbers of entrances into the enclosed arms were decreased, similar to those effects observed with diazepam. In the FST, acute treatment with VP, produced a dose-dependent decrease in immobility time, similarly to imipramine. VP also produced a significant dose-dependent decrease in the latency of sleeping time, while producing an increase in total duration of sleep; influenced memory consolidation of the animals only at lower doses, unlike those that produced anti-depressant and anxiolytic effects. In summary, the results suggest that VP presents several psychopharmacological activities, including anxiolytic, antidepressant, and hypno-sedative effects.

## 1. Introduction

Worldwide epidemiological studies since the 1970s indicate that about 30% of the adult population suffer from some form of sleep disorder [1, 2], and 15% to 25% are affected by episodes of anxiety, suggesting that this is a silent epidemic of the 21st century [3].

The use of medicinal plants for the symptomatic treatment of anxiety, insomnia, and various nervous disorders is well known and has increased significantly in recent years [4, 5]. The main plant drug used for these pathologies is *Valeriana officinalis* L., a European plant that is accepted by most international pharmacopoeias [6, 7], but is exotic to Mesoamerica, were it needs to be imported for clinical use. 

After ethnobotanical surveys, commercial availability in popular markets, and community-based primary healthcare groups, several species of so-called “valeriana” were identified, some belonging to the genus *Valeriana *[8, 9], and others to the Asteraceae or Poaceae family, some of them containing valepotriates [10]. After carrying out a nationwide survey, ten samples of *Valeriana prionophylla* Standl. (VP) were collected, selecting the plant that grows in Concepción Tututapa, as a possible equivalent to *V. officinalis*.

*V. prionophylla* is a perennial plant, which grows up to 10–80 cm in height, with small yellow or pale purple flowers in a long peduncle; the roots are 12–20 cm long, 4-5 cm wide, and it has a strong characteristic odor. It has been described growing in regions from Mexico to Costa Rica, at altitudes above 1500 m [11]. Of the scant studies on this plant species in the international literature, one demonstrates the presence of monterpenic iridoids (valerate, isovalerate, homovalerate, and didrovaltrate) [12], and another, its antioxidant and vasorelaxant activity, which is attributed to the presence of iridoids (7,9′:7′,9-diepoxylignan glycosides) [13]. National publications demonstrate that oral administration in mice does not show any signs of toxicity after administration for eight days (DL_50_ > 1,200 mg/kg) [14]; in 28 elderly patients with insomnia, a 1 : 5 tincture showed control of insomnia, and no secondary effects were observed [15]. Since this plant species is included in the official list of medicinal plants from Guatemala [16] and phytochemically seems to be very similar to the species *Valeriana officinalis,* the aim here was to demonstrate the behavioural and pharmacological activities observed in animals treated with ethanol extract of this specie.

## 2. Materials and Methods

### 2.1. Plant Material

Material was collected from cultivation in Tierra Blanca, Concepción Tutuapa, San Marcos (15°14.808′N, 91°55.430′W), Guatemala, a vegetation zone that belongs to a very humid low montane forest. Three-year-old rhizomes and roots were dug up, washed, and shade-dried. Botanical samples were determined by Mario Veliz at Herbarium BIGU, School of Biology, USAC, and a voucher sample deposited (no. 49183).

### 2.2. Preparation of Ethanol Extract

Dry material was ground, wet with 50% ethanol, and placed in a stainless steel percolator. 50% ethanol was added to obtain a tincture, which was concentrated in a rotavapor. Fresh ethanol was added for five consecutive days and the extract was concentrated. The final drying was performed in a vacuum dryer with silica gel [17]. The average yield of the extractable solids was 28.52%.

### 2.3. Drugs

Distilled water with 1 drop/ml of DMSO was used as vehicle. Diazepam (Cristália, Brazil) was used as the reference drug (positive control) for anxiolytic, sedative, muscle relaxant, and anticonvulsant activities. Pentylenetetrazol and strychnine (Sigma, USA; 4.0 mg/kg) were used to induce convulsions. Sodium pentobarbital (Sigma, USA; 50 mg/kg) was used to induce sleep; lorazepam (Cristália, Brazil) was used as positive control for hypnotic activity. Imipramine (Sigma, USA) was used as the standard drug for antidepressant effect, and phenobarbital (Sigma, USA) as the reference drug for anticonvulsant activity.

### 2.4. Animals

Nonfasted adult female Swiss mice (18–35 g) and male Wistar rats (150–220 g) were used in the experiments. The animals were kept in a controlled-temperature environment (22 ± 3°C), illuminated by daylight and supplemented with electric light from 7 : 00 a.m. to 7 : 00 p.m., with free access to food and water. The experiments were performed after approval of the protocol by the Institutional Ethics Committee of UNIVALI 3008/2007, and were carried out in accordance with the current guidelines for the care of laboratory animals, and the ethical guidelines for investigations in Brazil, COBEA (Brazilian College of Animal Experimentation) [18]. The number of animals and intensity of the noxious stimuli used were the minimum necessary to demonstrate the consistent effects of the drug treatments.

### 2.5. Measurement of Motor Performance: Rota Rod Test

To evaluate possible nonspecific muscle relaxant effects of VP extract, the mice were tested on the rota-rod [19]. The apparatus consists of a bar with a diameter of 2.5 cm, subdivided into six compartments by disks of 25 cm in diameter (Ugo Basile, Model 7600). The bar was rotated at a constant speed of 22 rpm. The animals were selected 24 h previously in order to eliminate those that did not remain on the bar for two consecutive periods of 60 sec. The animals were treated with VP extract (50–150 mg/kg, p.o.) or with the same volume of vehicle NaCl 0.9% solution with 2% of Tween 80, 60 min before the test. The results are expressed as the time in sec. that the animals remained on the rota-rod. The cut-off time used was 60 sec.

### 2.6. Open-Field Test (OF)

The open-field arena was made of acrylic (transparent walls and black floor). The arena measured 30 × 30 × 15 cm, divided into nine squares of equal areas. The open-field was used to evaluate the exploratory activity of the animals [20]. The animals received VP extract (50–150 mg/kg, p.o.) and vehicle 60 min prior to the tests. They were placed individually in the center of the arena, and allowed to explore freely. The observed parameters were: ambulation or crossing (the number of squares crossed with all four paws) and numbers of rearings, both indicators being recorded for the last 5 min of the 6 min testing period.

### 2.7. Pentobarbital-Induced Hypnosis

In order to evaluate hypnosis potentiating activity, sodium pentobarbital (50 mg/kg, i.p.) was injected into female mice after oral administration of VP extract (50, 100, and 150 mg/kg). The latency and hypnosis time were recorded. Hypnosis time was measured by the loss of the righting reflex, recovery of this reflex being considered as the end of the hypnosis time [21]. Lorazepam (2.0 mg/kg, i.p.) administered 30 min before the test was used as a reference drug.

### 2.8. Elevated Plus-Maze Test (EPM)

The procedure used to evaluate the possible anxiolytic effect was similar to that described previously [22, 23]. The wooden EPM apparatus was shaped in the form of a cross, with two open arms (50 × 10 cm^2^) with sides of 1 cm in height, and two closed arms (50 × 10 × 15 cm). The central area of the maze measured 10 × 10 cm^2^. The apparatus was elevated to a height of 70 cm. During the test, each animal was placed at the center of the maze facing the closed arm 60 min after administration of VP extract (50–150 mg/kg, p.o.) or vehicle. All entries to the open or closed arms were scored for 5 min, and the total time spent in each arm was recorded. An entry was defined as placing the mouse's four paws into an arm, and no time was recorded when the animal was in the center of the maze. Diazepam (0.75 mg/kg, i.p., administered 30 min before the test) was used as a reference drug.

### 2.9. Forced Swimming Test (FST)

The FST is the most widely used pharmacological model for assessing antidepressant activity [24]. This method is based on the observation that animals exposed to a situation of forced swimming became passive and immobile after a period of vigorous activity (struggling) and produced only enough movements to keep their heads above the water. The FST was carried out on mice according to Porsolt et al. [25]. Swimming sessions were conducted by placing the animals in an individual Plexiglass cylinder (46 cm high × 20 cm diameter) containing 20 cm of water at 24 ± 1°C. All the animals were forced to swim for 6 min. The amount of time spent in struggling (first 2 min of experiment) and immobile during the final 4-min interval of the test was manually recorded by competent observers. VP extract (50–150 mg/kg, p.o.), imipramine (10 mg/kg, i.p.) or vehicle was administered, 60 and 30 min before the test.

### 2.10. Pentylenetetrazole- (PTZ-) and Strychnine- (STR-) Induced Convulsions

Seizures were chemically induced with PTZ and STR, as described by Ali et al. [26] and Yamashita et al. [27] with minor modifications. Groups of 8–10 mice were treated with the VP extract (50–150 mg/kg, p.o.), phenobarbital (40 mg/kg, i.p.), or vehicle, and 30 and 60 min afterwards, they received a convulsant dose of PTZ (100 mg/kg, i.p.) or STR (4.0 mg/kg, i.p.). Immediately after treatment with the convulsant agents, the animals were placed individually in cages and observed for 60 min. The time of the onset of seizures, and mortality, were assessed during this time.

### 2.11. Inhibitory Avoidance (IA)

Rats were trained in a one-trial, step-down IA paradigm, a highly validated learning task in which stepping down from a platform present in a given context is associated with a foot shock resulting in an increase in step-down latency [28, 29]. The IA apparatus consisted of a 50 × 25 × 25 cm Plexiglass box with a 5 × 8 × 25 cm platform on the left end of a series of bronze bars that constitutes the floor of the box. During training, the animals were gently placed on the platform facing the left rear corner of the training box. When they stepped down and placed their four paws on the grid, they received a 2-second, 0.4 mA scrambled foot shock. Afterwards, they were immediately withdrawn from the training box. Memory retention was evaluated in a test session carried out 24 h after the training. In the test, the trained animals were placed back on the training box platform until they eventually stepped down onto the grid. The latency to step-down during the test session was taken as an indicator of memory retention. A ceiling of 180 sec was imposed for step-down latencies during the retention test. To evaluate the effect on memory consolidation, the treatment with VP extract (50–150 mg/kg, p.o.), or vehicle were given 60 min after training.

### 2.12. Statistical Analysis

The results are presented as mean ± SEM. For the parametric data, the variables were analyzed by one-way analysis of variance (ANOVA), followed by Student-Newman-Keuls analysis. *P* values <.05 were considered as indicative of significance. For the statistical analysis of non-parametric data from the IA test, the Mann-Whitney *U*-test was used.

## 3. Results

### 3.1. Rota-Rod Test

Neither of the doses of extract used, given p.o. 60 min beforehand, significantly affected the motor response of the animals when assessed in the rota-rod test. This effect was only observed in the animals treated with diazepam (data not shown).

### 3.2. OF

**Figure 1:**
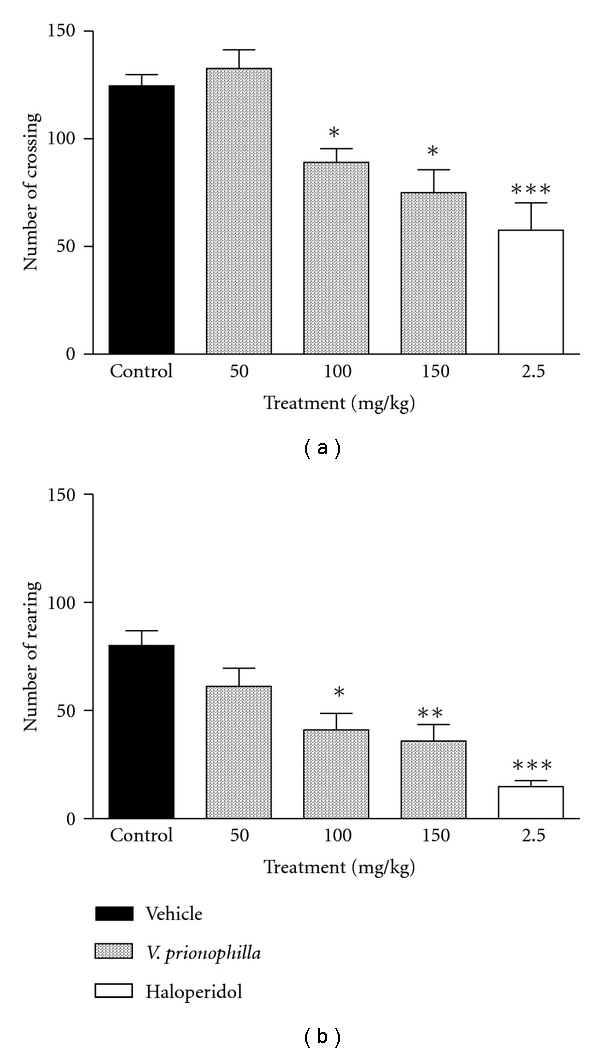
Effects of acute administration of hydroalcoholic extract of *Valeriana prionophylla* (50, 100 and 150 mg/kg, p.o.) and haloperidol (2,5 mg/kg, i.p.), in the open field test in mice. (a) Number of crossings and (b) number of rearings. Each bar represents the mean ± SEM, from 6 to 10 animals per group. *P < .05, **P < .01 and ***P < .001, compared with the corresponding control value. Data were analyzed by the ANOVA or *t*-test and complemented by Newman-Keuls *post hoc *test.

VP extract, at doses of 100 and 150 mg/kg, showed sedative effects in mice, as assessed by the OF (Figure 1). Significant effects were detected with both doses, which produced inhibition (28.57 and 39.80%, resp.) in the number of crossings (*P* < .05) compared with the controls (Figure 1(a)). The number of rearings (Figure 1(b)) was also significantly decreased with both doses, which produced percentages of inhibition of 48.75 and 56.25%, (*P* < .5, *P* < .01, resp.). Haloperidol, used as positive control, produced a statistically significant decrease in both parameters.

### 3.3. Pentobarbital-Induced Hypnosis

**Figure 2:**
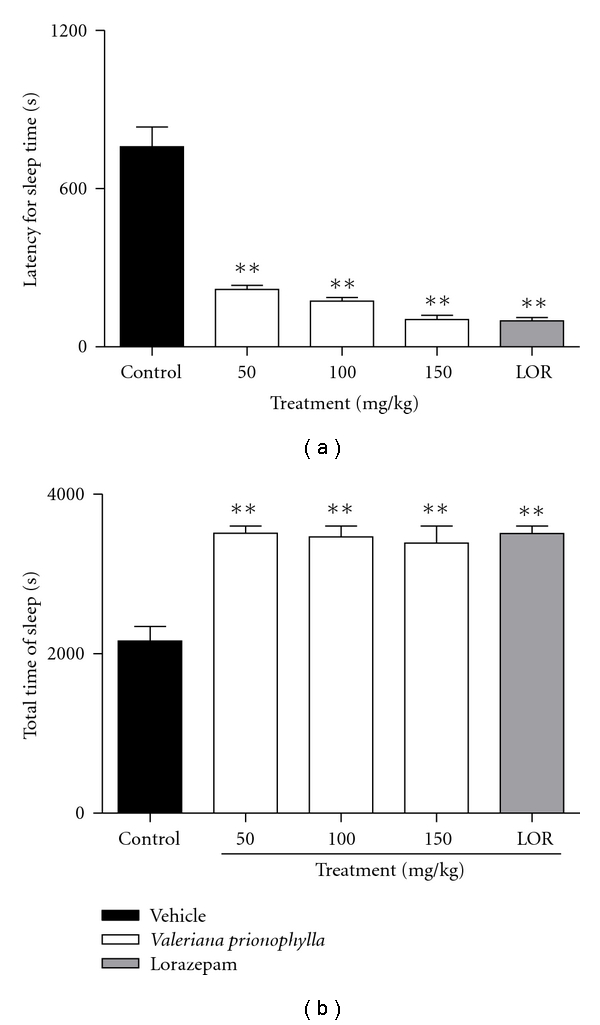
Effect of hydroalcoholic extract of *Valeriana prionophylla* (50, 100, and 150 mg/kg, p.o.) and lorazepam (2.0 mg/kg) on (a) time latency for sleep and (b) total sleep time induced by pentobarbital sodium (50 mg/kg, i.p.). Date are reported as means ± SEM. *N* = 8–10 mice **P < .01 compared to the control group (ANOVA followed by Student-Newmann-Keuls analysis).

In the barbiturate-induced sleeping time test, i.p. administration of VP extract 10, 50, and 100 mg/kg, respectively, decreased (*F* = 15.26) the latency sleep time by 71.62, 76.0 and 85.8%, respectively (Figure 2(a)). Treatment with VP extract (*q* = 6.362  *P* < .01, *q* = 6.141  *P* < .01, *q* = 5.778  *P* < .01) produced a statistical and dose-dependent increase in total sleep time (62.8, 60.6, and 57.0%, resp.) suggesting a potentiation of the pentobarbital effect (Figure 2(b)).

### 3.4. EPM

**Figure 3:**
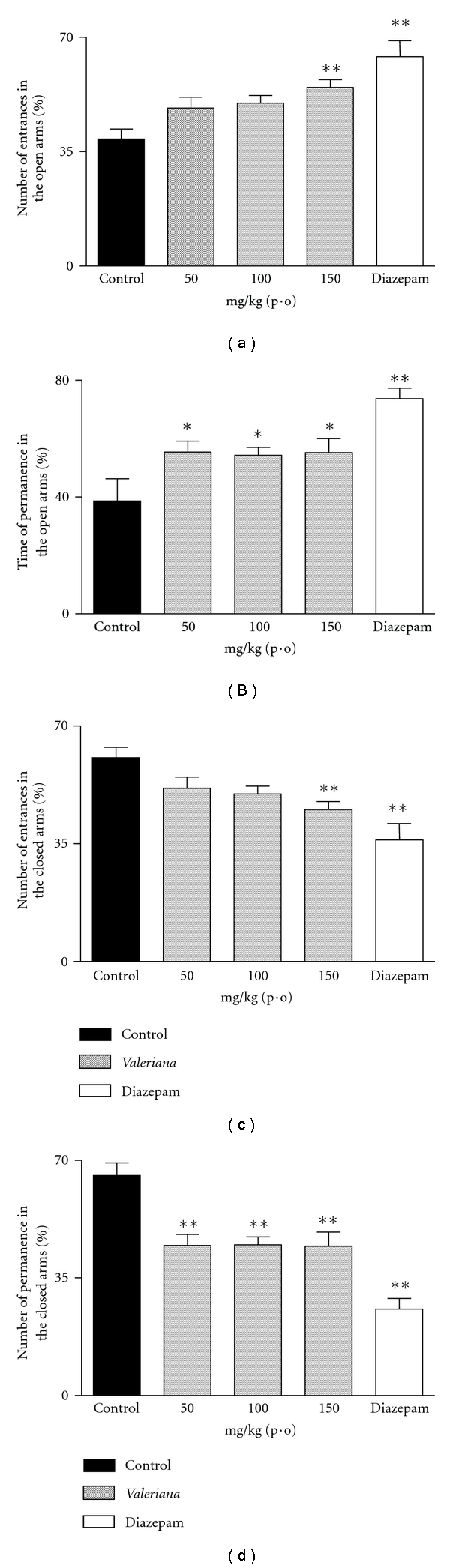
Effects of hydroalcoholic extract of *Valeriana prionophylla* (50, 100, and 150 mg/kg, p.o.) and diazepam (0.75 mg/kg, i.p.) in the elevated plus maze test, in mice. (a) Number of entrances to the open arms, (b) time spent in the open arms; (c) number of entrances to the closed arms, and (d) time spent in the closed arms. Each bar represents the mean ± SEM, from 8 to 10 animals per group *P < .05, **P < .01, compared with the corresponding control value. Data were analyzed by the ANOVA or *t*-test and complemented by Newman-Keuls *post-hoc *test.

A possible anxiolytic activity of VP was assessed by the EPM. The results (Figures 3(a) and 3(b)) showed that the extract (150 mg/kg, p.o.) promoted a modification in the number of entrances to the open arms (*q* = 3.326, *P* < .01) and the time spent in the open arms (*q* = 2.545, *P* < .05), which were significantly increased by 38.5 and 44.7%, respectively, compared with the controls. On the other hand, the same dose of VP extract (150 mg/kg, i.p.) promoted a decrease in the time spent and the number of entrances to the closed arms (Figures 3(c) and 3(d)), (*q* = 4.217, *P* < .01) of 32.5 and 25.60%. In this experiment, the anxiolytic effect of diazepam was also observed.

### 3.5. FST

**Figure 4:**
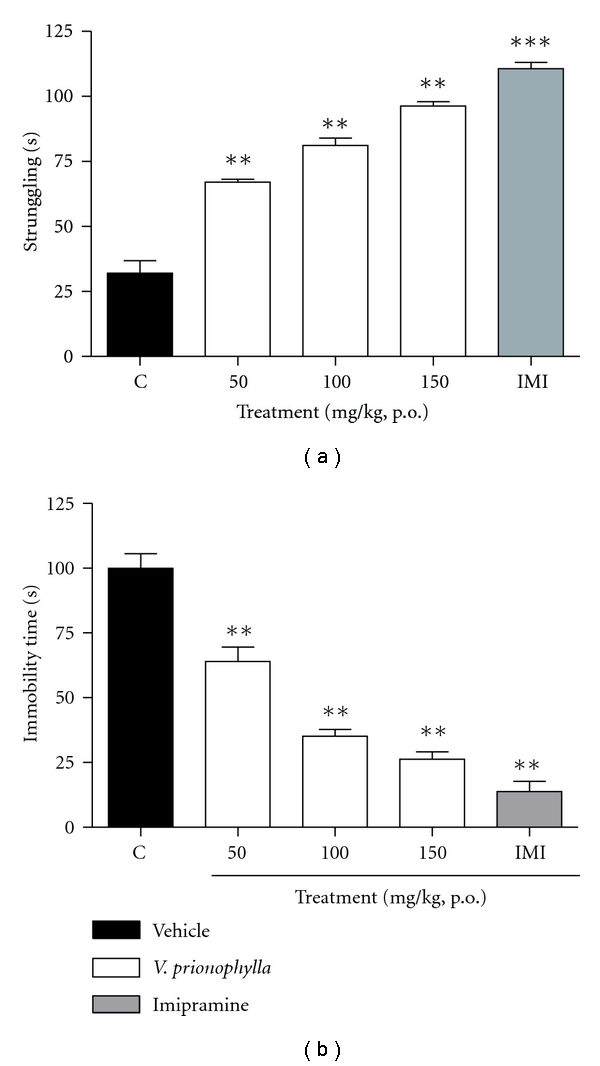
Effect of hydroalcoholic extract of *Valeriana prionophylla* (50, 100, and 150 mg/kg, p.o.) and imipramine (10 mg/kg, i.p.) with acute administration on (a) struggling and (b) immobility time in mice. Data are reported as means ± SEM. *N* = 8–10 mice. **P < .01, ***P < .001 compared to the control group (ANOVA followed by Student-Newmann-Keuls analysis).

As shown in Figure 4(a), it was observed that acute treatment with the extract exhibited a significant increase in the time spent struggling (*F* = 111.71, *P* < .01). This effect was observed in all the doses used (50, 100 and 150 mg/kg, p.o.). It was also observed that treatment with the extract promoted a significant increase in immobility time (*F* = 64.402, *P* < .01) in 35.60, 64.60, and 73.70%, compared with the controls (Figure 4(b)). The antidepressant effect of imipramine was observed in both parameters.

### 3.6. PTZ and STR-Induced Convulsions

Comparison between the treatment of animals with VP extract (50–150 mg/kg), phenobarbital (40 mg/kg) and the control group showed that only phenobarbital significantly suppressed STR- and PTZ-induced clonic seizures, with complete suppression, and was also able to protect against 24-hour lethality (results not shown).

### 3.7. IA

**Figure 5:**
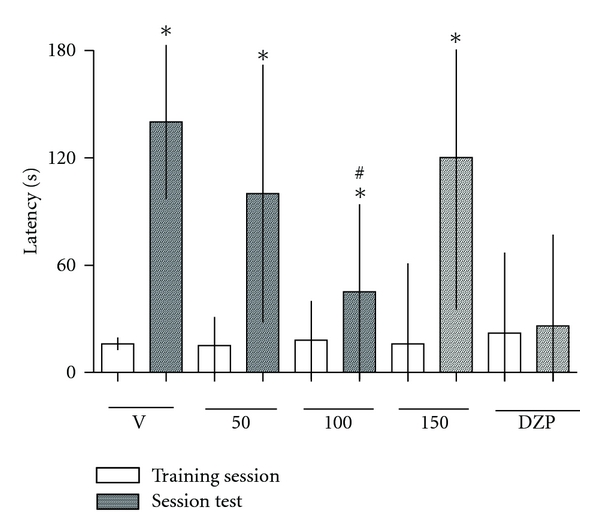
Effect of hydroalcoholic extract from *Valeriana prionophylla* (50, 100, and 150 mg/kg, p.o) and diazepam (0.75 mg/kg, i.p.) given immediately after training in a single trial, on memory retention of inhibitory avoidance measured 24 h later. Each bar represents the median (interquartile range) for 8 to 10 animals per group. *(*P* < .05) comparison between training and test sessions, ^#^(*P* < .05) compared with the corresponding control value in the session test only. Data were analyzed by the ANOVA test and complemented by the Kruskal-Wallis or Mann-Whitney *U*-tests.

Finally, for the VP extract, only the 100 (mg/kg, p.o.) dose produced inhibitory effects (*P* < .05) on memory retention in the animals treated in the inhibitory avoidance test (Figure 5).

## 4. Discussion

The central effects of VP were studied, and the results for the preliminary neuropharmacological screening showed that this extract exhibited sedative, hypnotic, anxiolytic, and antidepressant effects.

 In this study, the general depressant activity of VP extract was confirmed by a decrease in the sleeping latency and an increase in pentobarbital-induced sleeping time, which may be attributed to an inhibition of the pentobarbital metabolism or to an action in the regulation of sleep [30]. In addition to the lack of effect of VP extract in the animals submitted to the rota-rod test, VP also exhibited general depressant activity, though this activity did not interfere with the animals' motor system. Myorelaxant effects were not observed in dosages up to 1000 mg/kg in experiments using different extracts of *V. officinalis* [31].

The hypnotic action of pentobarbital was demonstrated by Petty [32] to be mediated by the GABA_A_ receptor complex. The decrease in rearing and crossing responses in the open field test confirms the depressant activity of VP, since it is conceded that rearing is a function of the excitability level of the central nervous system [33]. The results showed that VP was able to significantly decrease not only the number of crossings, but also the number of rearings. Since both parameters are indicators of locomotor activity, horizontal and vertical, respectively, and locomotor activity is considered as an index of alertness [34] the decrease these parameters indicates a sedative effect of VP.

In order to study the possible anxiolytic effect of VP extract, the EPM test was used. Anxiety, a symptom that accompanies various central nervous system disorders and is also a disorder in itself, is characterized in humans by a tense and exhaustive physical alertness [34]. Other animal species display a variety of defensive reactions in response to predators, some of which are understood as correlated states of anxiety [35]. Rodents demonstrate, aversion, fear, and curiosity when placed in a new environment, and an overall assessment of behavior can be determined through the observation of freezing, grooming (fear) or rearing, head-dips (curiosity), and the number of fecal boluses produced [22, 36].

 The EPM has been frequently used to detect and evaluate the anxiolytic/anxiogenic properties of drugs [37, 38]. The entries and time spent in the open arms is the main indicator of fear in the EPM, given the fact that an open area is extremely aversive to rodents [37, 39]. In addition, in the open arms there is no thigmotaxis [40] which enhances the state of fear and aversion in the animals. Our results showed that VP was also able to significantly increase the time spent, as well as the number of entries to the open arms, indicating a positive response in this test. These results also reinforce other data obtained in the recent literature, which show anxiolytic-like effects in more than one species of Valerian genus [21, 31, 41] making these plants a therapeutic option in the management of anxiety.

The FST has been validated as a suitable tool for predicting the anti-depressant properties of drugs [42–48]. The FST is a behavioral test that in rodents, gives an indication of the clinical efficacy of various types of antidepressant drugs. Nowadays, antidepressants are known to act by several distinct mechanisms at the receptor level, probably also stimulating similar pathways at the subcellular level [45]. The administration of VP prior to the test acute reduced total immobility time behavior. Several authors have proposed that immobility during the test could be an efficient adaptative response to this stress [25, 43, 44, 47]. VP affects the normal pattern of behavior during the test, suggesting an antidepressant behavior in response to an inescapable source of stress.

The effect of VP in memory consolidation was also examined. In the inhibitory avoidance test in particular, effects on memory were observed. Our results support the idea that VP interacts with the GABA_A_ receptor, probably at the level of the receptor subtypes that mediate BDZ effects, to produce the sedative and hypnotic activities observed. Sedative and anxiolytic drugs such as BDZs also have a propensity to cause cognitive impairment [49].

 The lack of effect of VP on PTZ- and STR- induced convulsions does not exclude the possibility of an anticonvulsant effect that could be achieved with higher doses of the extract or another type of animal model of seizure such as in the maximal electroshock convulsions or kindling. Recently Rezvani et al. [50] with doses far higher than those used in this study demonstrated anticonvulsant effect of aqueous extract of *Valeriana officinalis* in amygdala-kindled rats.

In the search for the active substances of *Valeriana officinalis*, many compounds have been isolated and identified during the last 20 years, but it is still uncertain which of them is responsible for the observed actions [50]. Concerning the chemical constituents of VP extract used in our experiments, classical phytochemical methods were used to determine the main constituents. The GC technique showed metabolites of interest, such as isovaleric, 3-methylvaleric, isovalerate allyl valeric, and 3-methyl-2-oxo valeric acids, glucuronide, and morphine 3-hydroxybromazepan. Two strands of research have attempted to indicate the chemical constituents responsible for the central effects of valerian. The first proposes that valerenic acids are responsible for the central effects while the second proposes that the flavonoids are responsible for these effects.

In animal experiments, valerenic acid or valerian extracts showed tranquilizing and/or sedative activity [51–57]. Valerenic acid has also been shown to modulate, or at high concentrations, activate the GABA_A_ receptors, as shown for recombinant receptors expressed in *Xenopus oocytes* [54, 55] or neonatal brain stem neurons [56]. Benke et al. [57] recently described the existence of a specific binding site on GABA_A_ receptors with nM affinity for valerenic acid and valerenol, common constituents of valerian species. Both agents enhanced the response to GABA in multiple types of recombinant GABA_A_ receptors. Besides the modulation of GABA_A_ receptors, several reports have shown the involvement of adenosine receptors in the effects of Valeriana [58–61].

In relation to flavonoids, several species of valerian (including *V. prionophylla*) present these compounds in their composition. Wasowski et al. [62] through a “ligand-searching approach” using purified extracts as far as possible, were able to report the presence of 6-methylapigenin in *Valeriana wallichii* DC and *V. officinalis *and to prove that it is a BDZ binding site (BDZ-bs) ligand, Marder et al. [41] reported the presence of 2S (−) hesperidin in *V. wallichii* and in *V. officinalis *and found that it has sedative and sleep-enhancing properties. 6-methylapigenin, in turn, proved to have anxiolytic activity and was able to potentiate the sleep-enhancing properties of hesperidin. Fernández et al. [21] reported the identification of the flavone glycoside linarin in *V. officinalis *and the discovery that it has, like hesperidin, sedative and sleep-enhancing properties that are potentiated by simultaneous administration of valerenic acid. 6-methylapigenin, another flavonoid was also identified in extracts of *V. officinalis,* and its effects on the central nervous system were detected [41]. However, the molecular mechanism of action of any of the valerian ingredients *in vivo* is not yet known. In our case, the studies are now in progress to determine the active principles of *V. prionophylla *responsible to the pharmacological effects demonstrated.
